# Time Is Money in Case of a Button Battery Ingestion

**DOI:** 10.1097/PG9.0000000000000259

**Published:** 2022-10-20

**Authors:** Charlotte Bosschaert, Katrien Van Renterghem, Dirk Van de Putte, Lucas Matthyssens, Saskia Vande Velde, Pauline De Bruyne, Ruth De Bruyne, Emma Beel, Stephanie Van Biervliet

**Affiliations:** From the * Department of Pediatrics, Ghent University Hospital, Ghent University, Ghent, Belgium; † Department of Pediatric Surgery, Ghent University Hospital, Ghent University, Ghent, Belgium; ‡ Department of Paediatric Intensive Care, Ghent University Hospital, Ghent University, Ghent, Belgium.

**Keywords:** foreign body ingestion, pediatrics, dysphagia, esophageal perforation

## Abstract

Button battery (BB) ingestion is a preventable pediatric health hazard with important morbidity and mortality due to complications. We present 3 pediatric patients with a complicated course after BB ingestion and discuss current guidelines. Urgent endoscopic removal is necessary for every BB impacted in the esophagus. A new strategy before endoscopic removal is the administration of honey or sucralfate. During endoscopy, rinsing the esophageal mucosae with acetic acid can neutralize the alkalic environment and prevent late complications. Prevention of ingestion needs to be pursued by increasing awareness and changing legislation of packaging of BB.

## INTRODUCTION

The frequency of presentation to the emergency room after unintentional foreign body ingestion in children doubled over the past 13 years (1). The ingestion of a button battery (BB) is feared due to associated complications (2). A 7-fold increase of these complications over the past 2 decades, places BB ingestion high on the list of preventable pediatric health hazards (3). Raising awareness among caregivers and general public is essential in the prevention and correct handling of these accidents.

Morbidity and mortality as a result of BB ingestion are mainly the result of esophageal impaction (2). The alkaline esophageal environment increased size and power of the BBs and younger age of the child are associated with an increased esophageal impaction risk (2,3). The risk for severe complications further increases with the duration of impaction and remaining voltage of the BB (4).

Based on 3 complicated cases over the past 6 months, presentation, diagnosis, and new guidelines will be discussed.

## CASE REPORT

### Case 1

**FIGURE 1.**
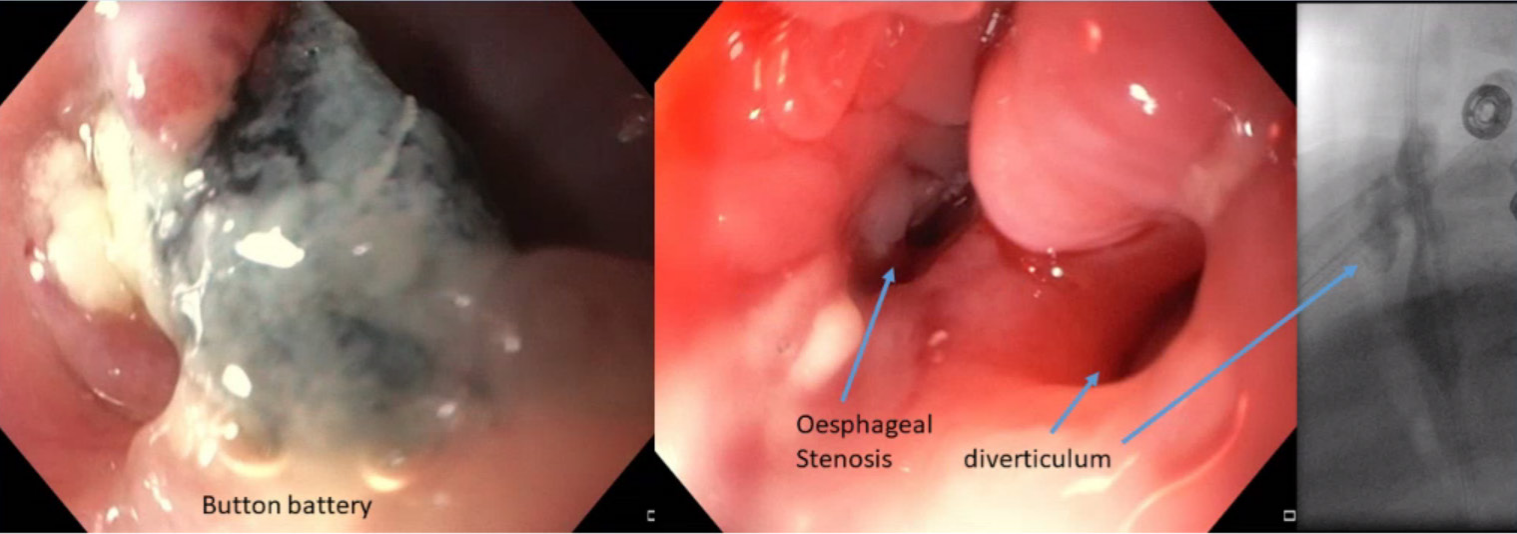
Case 1: endoscopic view before and after button battery removal. Result of the esophagogram.

A previously healthy 20 months old girl presented with dysphagia for 6 months. She was only able to drink or eat mixed foods, but no solids. She suffered from chronic rhonchi starting in the same period. Physical examination showed no respiratory distress. There were stagnating saliva in the throat and a poor weight gain since 6 months. Gastroscopy revealed a BB below the upper esophageal sphincter. After BB removal, a food-containing diverticulum and distal stenosis were visualized. She was tube fed, received antibiotics and a proton pump inhibitor (PPI). The diverticulum was confirmed on an esophagogram (Fig. 1). She was discharged after 2 weeks with tube feeding. It remained necessary until the esophageal diameter allowed liquid and pureed food to pass. Repeated endoscopic dilatations and an attempt to endoscopically treat the diverticulum were insufficient to resolve the feeding problems, therefore surgery will be planned.

### Case 2

**FIGURE 2.**
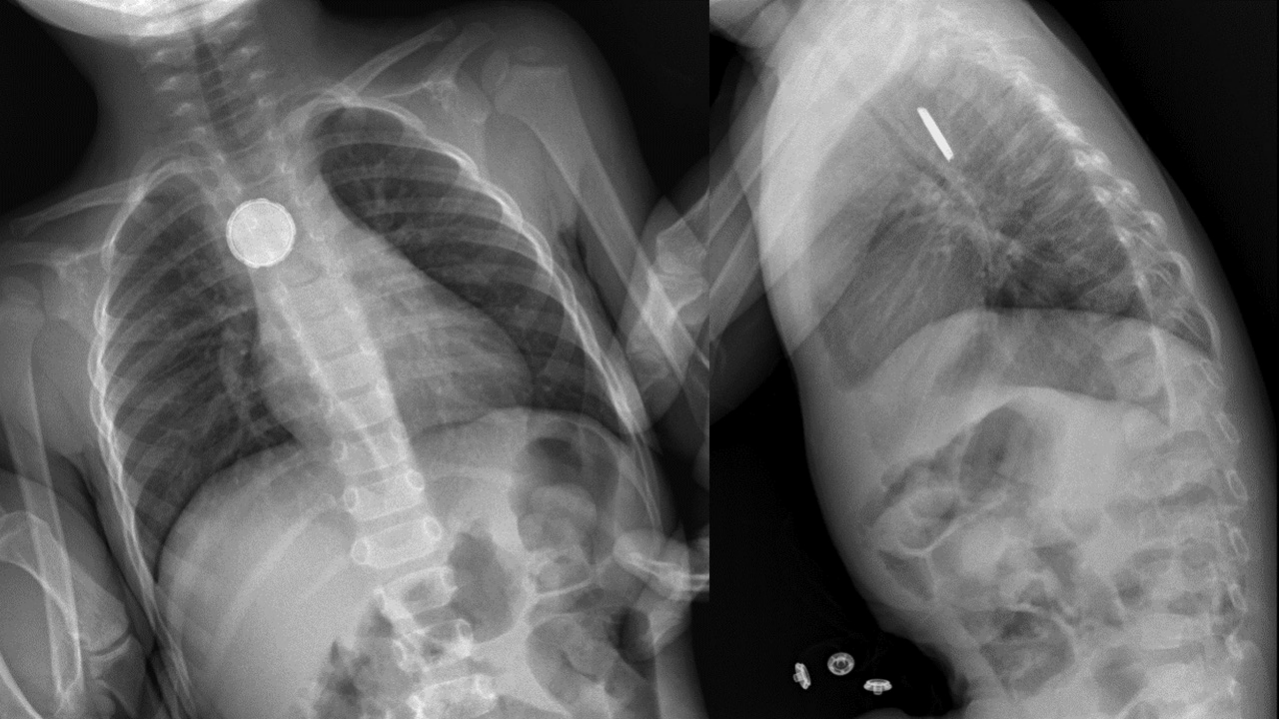
Case 2: radiograph revealing the presence of a button battery with typical halo sign and posterior step-off in lateral view.

An ill-looking 2-year-old boy presented with fever and dysphagia for solids. He was treated with antibiotics for otitis media. During hospitalization, he deteriorated with pallor and grunting. Laboratory studies revealed increased C-reactive protein (180 mg/L). Thorax radiograph showed a foreign body in the esophagus with a double halo sign on anterior-posterior view and a “step-off” sign on lateral view, that is, a BB (Fig. 2). Computed tomography showed no esophageal perforation but arguments for mediastinitis. Endoscopic removal of the BB was performed and showed necrosis at the esophageal mucosa. Tube feeding was started. Pharmacological therapy consisted of continuing broad-spectrum antibiotics and starting PPI. An esophagogram 2 days later showed a minor perforation. After 1 week tube feeding, oral intake was resumed with pureed and liquid food and discharge after 2 weeks. Endoscopy 6 weeks later showed a non-stenotic esophageal scar.

### Case 3

A 16-month-old girl ingested a foreign body. It was described as a coin on the radiograph. Endoscopic removal 8 hours after ingestion revealed a BB with mucosal injury. One day later, she developed fever with biochemical signs of infection. Computed tomography thorax showed a pneumonic infiltrate without signs of pneumomediastinum. Treatment with antibiotics resulted in clinical improvement. One week after BB removal, the patient presented with drooling and food refusal. Endoscopy revealed a tracheoesophageal fistula. Surgical repair of the tracheal perforation with sternocleidomastoid muscle and suture of the esophageal perforation was performed. She remained ventilated for 8 days and was treated with PPI and broad-spectrum antibiotics for 3 weeks for mediastinitis. She had paresis of the vocal cords, possibly due to recurrent laryngeal nerve injury. An esophagogram after 3 weeks showed a relapse of tracheoesophageal fistula which closed spontaneously. After 1 month, fluid intake was resumed and discharge home was possible after 5 weeks of hospitalization. This episode was followed by repeated endoscopic dilatations with 1 session of steroid injection.

## DISCUSSION

**FIGURE 3.**
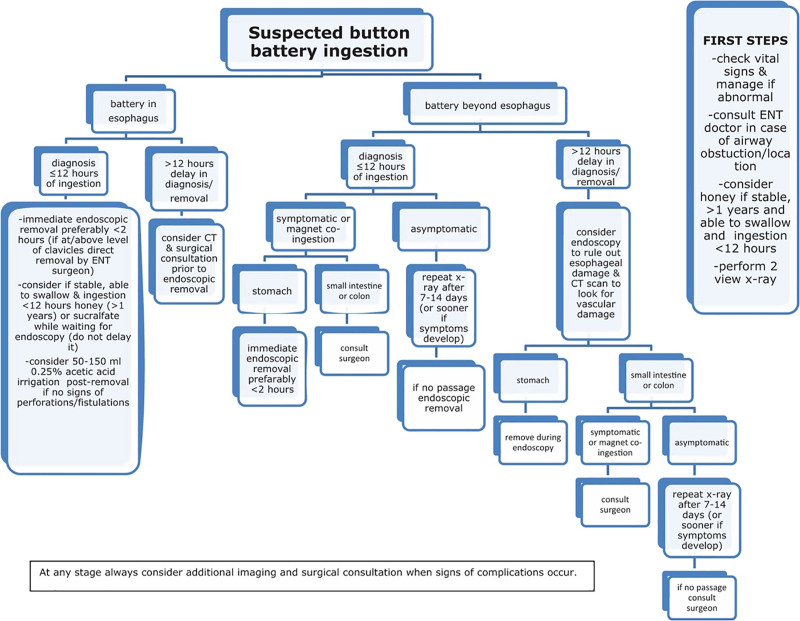
Diagnostic and therapeutic algorithm for button battery ingestions (4). CT, computed tomography; ENT, ear, nose and throat surgeon.

As demonstrated by the clinical cases, timely diagnosis and treatment are essential to avoid complications. Current guidelines include new strategies before BB removal and during endoscopy to prevent complications. The authors provide a flow chart for suspected BB ingestion (Fig. 3) (4).

During the time-lapse between ingestion and removal, the impact of a recent BB ingestion (<12 hours) in children can be limited using honey (above 12 months of age) or sucralfate (1 g/10 mL) (5). The advised dose for both is 10 mL (2 teaspoons) every 10 minutes with a maximum of 6 doses of honey and 3 doses of sucralfate (4). If no signs of perforation are present, this strategy can be explained to parents when they call the emergency ward. It should never be a reason to delay the endoscopy and the child should remain otherwise nil per os (6).

As symptoms are only present in 30% of patients, they cannot be used to decide whether or not investigations are indicated (7,8). However, severe symptoms (fever, hematemesis, stridor, hoarseness, back pain) might be indicative of complications (4). The halo sign (double ring) on radiograph is a well-described item to recognize a BB (8). A lateral radiograph makes it possible to locate the negative pole (the step-off site) and therefore to anticipate possible complications (4). In case of a delayed diagnosis (>12 hours) (9) or symptoms compatible with complications, it is recommended to perform a computed tomography scan before removal, to evaluate possible complications (4). In case of symptoms after removal of the battery, an magnetic resonance imaging scan can shed light on the underlying complication (4).

As mucosal damage can occur within 2 hours after ingestion, immediate endoscopic removal is necessary for every BB impacted in the esophagus, even if the patient has eaten (4). During endoscopy, good inspection and localization of the negative pole improves the complication risk assessment. Based on small series, neutralization of the remaining hydroxide with acetic acid irrigation (50–150 mL 0.25% acetic acid [= 8 mL acetic acid 3% in 92 mL sterile water]) might reduce late complications. It is only advised in the absence of perforation (10). The majority of batteries located beyond the esophagus will pass spontaneously within 7–14 days. Radiograph tracking of the BB progression is advised in asymptomatic patients, unless the battery is detected in the stool. In case of a delayed diagnosis, endoscopic evaluation of the esophagus is advised as it is unknown how long the BB took to transit through the esophagus. Depending on the localization, endoscopic or surgical removal of the BB is recommended if there is no progression or if the patient is experiencing symptoms (4).

All patients with mucosal damage should be admitted for monitoring. A normal esophagogram after 1–2 days gives the green light for a liquid diet which, if well tolerated, can be expanded to soft food for another 4 weeks. A second look endoscopy after 2–4 days might be considered as it provides prognostic information (11). In case of severe mucosal injury or perforation, broad-spectrum antibiotics are indicated to treat mediastinitis (4). The use of PPIs has not been studied.

The prevention of these major complications can only be obtained with increased awareness of public and health professionals and changing measures by the government and the industry (10,12). Of all BB ingestions, 70% can be avoided with screw secured compartments, individual blisters, bitter-tasting BB, and covering one side of a BB (12).

## CONCLUSION

BB ingestion is a preventable pediatric health hazard with increasing prevalence. Due to fistulization to surrounding tissues, complications are the main cause of morbidity and mortality. BB impaction in the esophagus is an emergency and requires urgent endoscopic removal. New strategies to prevent mucosal damage include administration of honey or sucralfate before endoscopic removal. During endoscopy, acetic acid irrigation of the esophageal mucosa might reduce late complications. Prevention by adjusting packaging and secure compartments needs to be pursued by legislation.
